# Decomposing the space of protein quaternary structures with the interface fragment pair library

**DOI:** 10.1186/s12859-014-0437-4

**Published:** 2015-01-16

**Authors:** Zhong-Ru Xie, Jiawen Chen, Yilin Zhao, Yinghao Wu

**Affiliations:** Department of Systems and Computational Biology, Albert Einstein College of Medicine of Yeshiva University, 1300 Morris Park Avenue, Bronx, NY 10461 USA

## Abstract

**Background:**

The physical interactions between proteins constitute the basis of protein quaternary structures. They dominate many biological processes in living cells. Deciphering the structural features of interacting proteins is essential to understand their cellular functions. Similar to the space of protein tertiary structures in which discrete patterns are clearly observed on fold or sub-fold motif levels, it has been found that the space of protein quaternary structures is highly degenerate due to the packing of compact secondary structure elements at interfaces. Therefore, it is necessary to further decompose the protein quaternary structural space into a more local representation.

**Results:**

Here we constructed an interface fragment pair library from the current structure database of protein complexes. After structural-based clustering, we found that more than 90% of these interface fragment pairs can be represented by a limited number of highly abundant motifs. These motifs were further used to guide complex assembly. A large-scale benchmark test shows that the native-like binding is highly likely in the structural ensemble of modeled protein complexes that were built through the library.

**Conclusions:**

Our study therefore presents supportive evidences that the space of protein quaternary structures can be represented by the combination of a small set of secondary-structure-based packing at binding interfaces. Finally, after future improvements such as adding sequence profiles, we expect this new library will be useful to predict structures of unknown protein-protein interactions.

**Electronic supplementary material:**

The online version of this article (doi:10.1186/s12859-014-0437-4) contains supplementary material, which is available to authorized users.

## Background

Interactions between proteins dominate all major biological processes in living cells [1-3]. Through these interactions, proteins either form permanent complexes such as supramolecular machines [4], or undergo transient binding such as their participation in cell signaling pathways [5]. The thermodynamics and kinetics of protein-protein interactions (PPI) are largely determined by the properties of their interfaces, where proteins make direct physical contacts. Therefore, deciphering the structural features of interacting proteins, especially at their interfaces, is a crucial step towards understanding the molecular organizations of cells [6,7]. Structural modeling of PPI is generally classified into two categories [8]. Traditional docking methods only rely on the geometric and chemical-physical complementarity of modeled protein surfaces [9-11]. These methods (template-free methods) explore all possible binding modes of two proteins without a priori knowledge of their complex structures. In contrast, a number of recent studies have used structurally characterized complexes as templates to construct models of unknown PPI [12-17]. These methods are called template-based methods. As template-free methods are limited by the ability of sampling the entire conformational space, template-based methods are facing difficulties with limited number of complex structures in current Protein Data Bank (PDB). Interestingly, these template-free and template-based methods of PPI modeling correspond to the *ab initio* [18] and homology modeling [19] in protein structure prediction. In protein structure prediction, there is also a third class of method, called fragment assembly which combines *ab initio* sampling and templates of protein fragments in PDB [20]. Considering the remarkable success of fragment-based methods in predicting protein tertiary structures [21], it is reasonable to anticipate that the similar idea can be extend to model PPI, in additional to the template-free and template-based methods.

In terms of modeling the structures of PPI, there is a further question regarding the nature of PPI space: whether proteins adopt a finite number of quaternary structures [22]. Similar question was asked about protein tertiary structures. Packing of secondary structural elements in protein tertiary structures is preferential [23,24]. It was estimated that there are approximately 1000 types of structural folds in the space of protein domains [25]. Despite the fact that protein fold space is regarded as rather continuous and multidimensional [26,27], it was found that a surprisingly small set of super-secondary structural elements (Smotifs) is sufficient to describe all known folds [28]. Moreover, novel folds are resulted from a new combination of existing Smotifs. A dictionary of tertiary structural motifs was also constructed to describe a substantial portion of protein structure space [29]. On the level of protein quaternary structures, a simple alignment method was recently applied to study the structural similarity of representative protein–protein interfaces [30]. It was found that the structural space of protein–protein interfaces is highly degenerate, where 80% of the interfaces form a dense network [31]. This indicates the importance of decomposing the space of protein–protein interfaces into smaller fragments, giving the potential usage of fragment-based method in modeling PPI as discussed in the previous paragraph.

**Figure 1:**
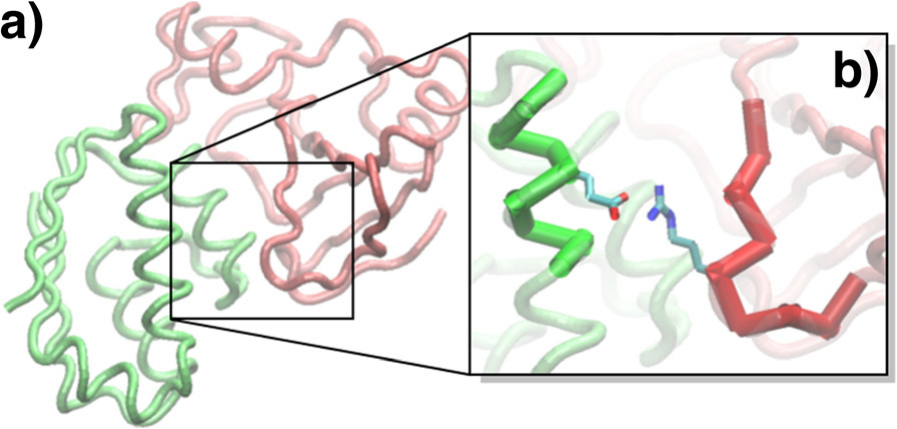
**We collected information of interface fragment pairs from a database of interacting protein domains.** For a pair of two protein domains that form physical contacts **(a)**, an interface fragment pair is defined as a pair of 9-amino-acid-long fragments, in which their centered residues form at least one atomic contact **(b)**.

Here we constructed an interface fragment pair library from the current structure database of protein complexes. For any dimeric complex in the database, an interface fragment pair is defined as a pair of 9-residue-long fragments from each side of the complex. Both residues in the middle of these two fragments are located at the dimer interface and form contacts at the atomic level (Figure 1). All pairs of fragments from all complexes in the database were recorded by the coordinates of their C-α atoms. Pairs with similar packing geometry were then clustered together. Only clusters that contain a relatively large number of fragment pairs were selected. The library consists of representative structures of all these most abundant clusters. We further used the library to guide complex assembly by aligning all structurally similar fragments from the two monomers to the corresponding fragment pair in the library. Through the test on a large-scale protein docking benchmark [32], we found that native-like quaternary structures were among all assembled complex models with a successful rate of more than 90%. Our study indicates that the structural space of PPI can be decomposed by a limited number of interacting fragments. Furthermore, after adding more features such as sequence profiles in the future, we expect this new library will be proved useful in predicting quaternary structures of unknown PPI.

## Methods

**Figure 2:**
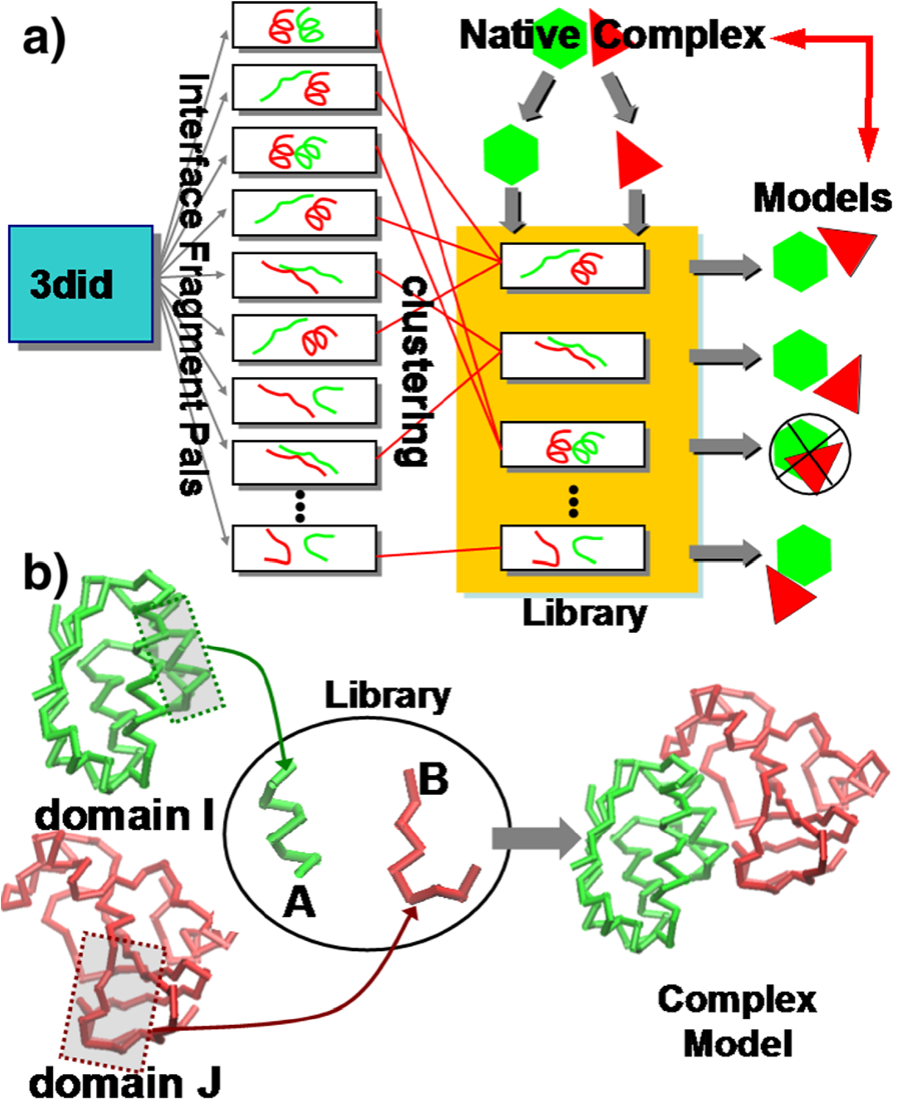
** The overall procedure of the method.** The flowchart of systematically constructing and testing the interface fragment pair library is shown in **(a)**. We first collected all pairs of interface fragments from the 3did database. These data were then clustered. A subset of recurrent structures were further selected as the library. Finally, the library was used to guide complex assembly. During the complex assembly, we assigned a sliding window along each protein domain. The Cα atoms in the windows were aligned to each fragment pair in the library **(b)**. If RMSDs are smaller than the cutoff value, we further superimposed the structures of entire domains to their corresponding fragments according to the relative position of windows in each domain. After both domains were aligned to the interface fragment pair, a dimeric complex was constructed.

The systematic construction and test of the interface fragment pair library presented here was performed in three basic steps (Figure 2a). First, we collected all pairs of interface fragments from the database of 3D Interacting Domains (3did) [33]. We then clustered these data and selected a subset of recurrent structures as the interface fragment pair library. Finally, the library was used to guide complex assembly.

### Collecting information of interface fragment pairs from 3did database

For a given pair of protein domain I and J that form physical contacts (Figure 1a), we started the data processing by detecting all the residues at the binding interface. If a residue in domain I interacts with any residue in domain J, this residue was designated as an interface residue. Vice versa, if a residue in domain J interacts with any residue in domain I, it was designated as an interface residue. For all residues at the interface, we generated a list of interface residue pairs. Any pair in the list has to meet two criteria: 1) one residue in the pair comes from protein domain I and the other comes from J; 2) after calculating distances of all side-chains atoms between two residues in the pair, the distance of at least one inter-residue atomic pair should be smaller than the cutoff value that equals to 5 Angstrom (Figure 1b). The information of local backbone conformation was further taken into account for each residue pair in this list. A window of 9 amino acids that is centered at the corresponding interface residue was assigned for both sides of the pair (red and green fragments in Figure 1b). The local conformation is represented by the coordinates of Cα atoms for these two fragments. Consequently, with these local Cα coordinates, the interface residue pair becomes the interface fragment pair. It is worth mentioning that, given two fragments from two interacting domains, one fragment is from residue 1 to 9 of domain I, while the other fragment is from residue 101 to 109 of domain J. If residue 5 of domain I forms interactions with both residue 105 and 106 of domain J, these interactions will be recorded twice. In this specific case, they are identified as two fragments in domain J. The first record is centered at residue 105 and the fragment is from residue 101 to 109 of domain J. The second record is centered at residue 106 and the fragment of domain J is from residue 102 to 110.

The list of all interface fragment pairs for a given PPI was generated by above procedure. Following the same procedure, we collected data from 3did database. The 3did database selects a large number of domain-domain interactions in proteins for which high-resolution three-dimensional structures are available. The database consists of a large group of items called interacting domain pairs (ID). The interacting domain pair could be homodimer, heterodimer, or inter-domain interaction within a single subunit. Information about Pfam index is given for both domains of an ID. Each ID further includes different number of instances. The specific instances are called 3D items in which information about PDB index, chain id and residue range are provided for both interacting protein domains. In order to construct the interface fragment pair library, we selected only one representative 3D item from each ID in 3did database to reduce redundancy. For each selected 3D item, all interface fragment pairs were extracted from the structure of domain-domain interaction by the algorithm described in the previous paragraph. Finally, a total number of 153127 entries were derived.

### Constructing the interface fragment pair library by clustering the collected data

All the interface fragment pairs collected from 3did database were structurally clustered by the following simple algorithm. An initial fragment pair was randomly picked from all the entries and was set as the first cluster. We then further selected another entry randomly from the same pool. For this second entry, structure alignment was carried out with the first cluster. Root mean square difference (RMSD) was calculated after the alignment for all the Cα atoms in both fragments between the first and the second selected entries. If the RMSD was smaller than a predetermined cutoff value, the second entry was merged into the first cluster. Otherwise a new cluster was created for the second entry. Similar procedure was iterated for all the rest entries. Assuming before the *i*^th^ picked entry enters the clustering algorithm, the previous *i-1* entries form *m* clusters. We will compare the *i*^th^ entry with all the previous *i-1* ones and find the nearest neighbor that has the smallest RMSD with the *i*^th^ entry. If the RMSD value is smaller than the cutoff, the *i*^*th*^ entry will be assigned to the same cluster as its nearest neighbor belongs to. Otherwise we will generate the m + 1 cluster for this entry. For simplification, we selected a member from each cluster as the representative model of the cluster. The representative model has the most number of neighbors with the other members in the corresponding cluster.

The cutoff value of RMSD during clustering was empirically adopted. If the cutoff is too low, there will be too many clusters and the library will lose generality. On the other hand, if the cutoff is too high, pairs without structural similarity will be classified into the same group so that the library will lose accuracy. The current cutoff value we used in this study equals to 4.0 Angstrom. This leads to 2135 clusters from original 153127 interface fragment pairs. Clustering using other cutoff values will be discussed in the results. Moreover, the purpose of constructing the library is to decompose the space of protein quaternary structures into lower dimensions. Therefore, only the most representative clusters in which the number of members is higher than a cutoff value were selected into the library. The cutoff value was also empirically determined. If the cutoff is too high, we will lose the coverage for the structural space of fragment pairs. In contrast, if the cutoff is too low, the application of the library will be limited by the large number of clusters. In this study, any clusters with larger than 20 members was selected. Finally, the library includes 459 clusters and each cluster is represented by a structural model of the corresponding interface fragment pair.

The stability of our clustering process was tested across different runs. Specifically, five independent clustering runs were carried out. Each run was generated by a random order. The clustering results are listed in Additional file 1: Table S1 of the supplemental document. All five runs ended up with very close number of total clusters. The numbers of the most abundant clusters which contain more than 20 members are also very close among these five runs. Moreover, clusters in all runs were ranked by the number of their members. The ranking profiles are plotted as Additional file 1: Figure S1 in the supplemental document. The high similarity of these profiles suggests that the topology of clusters does not change between different runs. These testing results indicate the stability of our clustering process. In order to increase the robustness of the clustering result, future improvement of our method includes the application of hierarchical clustering algorithms, for instance, the single-linkage clustering algorithm.

### Complex assembly guided by the interface fragment pair library

Given structures of any two interacting protein domains I and J we explore all possible modes of their binding based on the constructed interface fragment pair library. Without a priori knowledge of binding sites for both domains, we assume that any residue pair from both of their surfaces can be located on the binding interface. Therefore, we first enumerate all potential combinations of fragment pairs in the complex by assigning 9-amino-acid long windows for both domains. The 9 consecutive residues in the window correspond to a fragment in the tertiary structure and the window can slide from N to C terminus. Assuming the two protein domains contain N_I_ and N_J_ residues, respectively, the total combination numbers of fragment pairs is (*N*_*I*_ − 8) × (*N*_*J*_ − 8). Under each combination, if the center residues of both fragments are on the protein surfaces, we further compare the structures of these two fragments to all the 459 entries in the interface fragment pair library. The surface residues are defined as any residue with solvent accessible surface area larger than 10 Ǻ^2^, which was calculated with atomic details of protein structures using a probe size of 1.4Ǻ.

If a specific entry in the library includes an interface pair of fragments A and B, we first align the Cα coordinates of residues in the current window of domain I to the fragment A and align residues in the other window of domain J to fragment B. In parallel, we align residues in the window in domain I to fragment B and align window in J to A. In either case, we calculate the RMSD for both alignments. If both RMSD values are smaller than 2Ǻ, we further superimpose the structures of entire domains I and J to their corresponding fragments A or B according to the relative position of windows in each domain (Figure 2b). After both domains are aligned to the interface fragment pair, a dimeric complex is constructed. The above process is iterated through all entries in the library for all (*N*_*I*_ − 8) × (*N*_*J*_ − 8) combinations of fragment pairs. This leads to an ensemble of structural models for binding between protein domains I and J. Structural models include inter-residue clashes are eliminated from the ensemble. We hypothesize that this derived ensemble forms a representative space of quaternary packing between two protein domains of known structures. We will evaluate the likelihood of the native binding mode being in this space by a large-scale benchmark test.

## Results and discussion

### The statistics of interface fragment pairs in database of interacting domains

**Figure 3:**
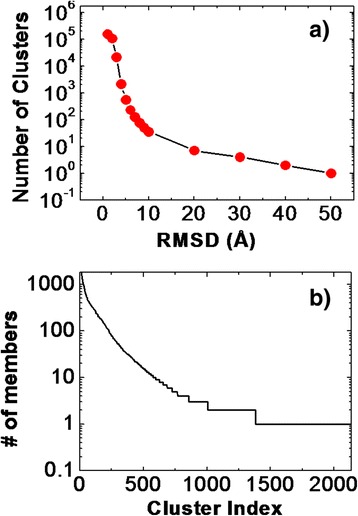
**We clustered all the 153127 interface fragment pairs in the 3did database using different values of RMSD cutoff.** The derived numbers of clusters under different RMSD values are plotted in **(a)**. Based on these statistical results, a cutoff value of 4 Angstrom was chosen in the following studies. This results in a totle number of 2135 clusters. We counted the number of fragment pairs in these 2135 clusters and ranked them in decreasing order **(b)**. We show that fragment pairs are not uniformly distributed in all clusters. A small number of clusters are highly abundant. As a result, only clusters with more than 20 members were considered, leading to the library of 459 highly representative entries. These 459 clusters cover more than 90% interface fragment pairs from the whole database.

We collected 153127 interface fragment pairs from 3did, a database of interacting protein domains. These interface fragment pairs belong to 4960 interacting domains. Each interacting domain is under one specific ID of 3did. The interactions are either formed as homo-dimer or hetero-dimer by different proteins, or formed by different domains in a same protein. The 153127 pairs were classified based on their structural similarity. The criteria of structural similarity are based on calculating the RMSD of Cα atoms between the two comparing fragment pairs. In this study, the cutoff value of RMSD was given empirically. Consequently, the results of classification depended on the determination of this RMSD cutoff. In order to systematically test the RMSD dependence of clustering results, we changed the cutoff values from 1Ǻ to 50Ǻ. For each cutoff value, clustering was carried out over all the 153127 pairs in the database. Figure 3a gives the derived number of clusters under different value of RMSD cutoff. The plot shows that the total number of clusters decreases fast when the cutoff value becomes larger. When the cutoff equals to 1Ǻ, there are 153127 clusters, indicating that no more than one interface fragment pairs can be clustered into the same group. This gives the minimal resolution for the structural difference between interface fragment pairs. When the cutoff equals to 4Ǻ, the cluster number reduces to 2135. Finally, when the cutoff increases to 50Ǻ, there is only one cluster. This indicates that all pairs were clustered into the same group, suggesting that clustering will lose sensitivity under large cutoff value. Based on these statistical results, a cutoff value of 4Ǻ was chosen in the following studies.

After classification, we further analyzed the distribution of interface fragment pairs in different clusters. We counted the number of fragment pairs in each of the 2135 clusters that were generated with a RMSD cutoff of 4Ǻ. All clusters were further ranked in decreasing order of pair numbers. Figure 3b gives the statistics of our calculated results. The x axis of the figure is the cluster index after ranking, while the y axis is the logarithm scaled number of interface fragment pairs in each corresponding cluster. The figure indicates that fragment pairs are not uniformly distributed in all clusters. A small number of clusters are highly abundant. For an example, the most abundant cluster consists of 15884 members, which hold about 10% of all fragment pairs. In contrast, a large number of fragment pairs are not frequently observed in database. For instance, there are 754 clusters only including one member. In order to construct a library containing the most representative structures of interface fragment pairs, we removed clusters that are not well abundant. As a result, only clusters with more than 20 members were considered, leading to the library of 459 highly representative entries. These 459 clusters cover more than 90% interface fragment pairs from the whole database.

### The structural features of interface fragment pair library

**Figure 4:**
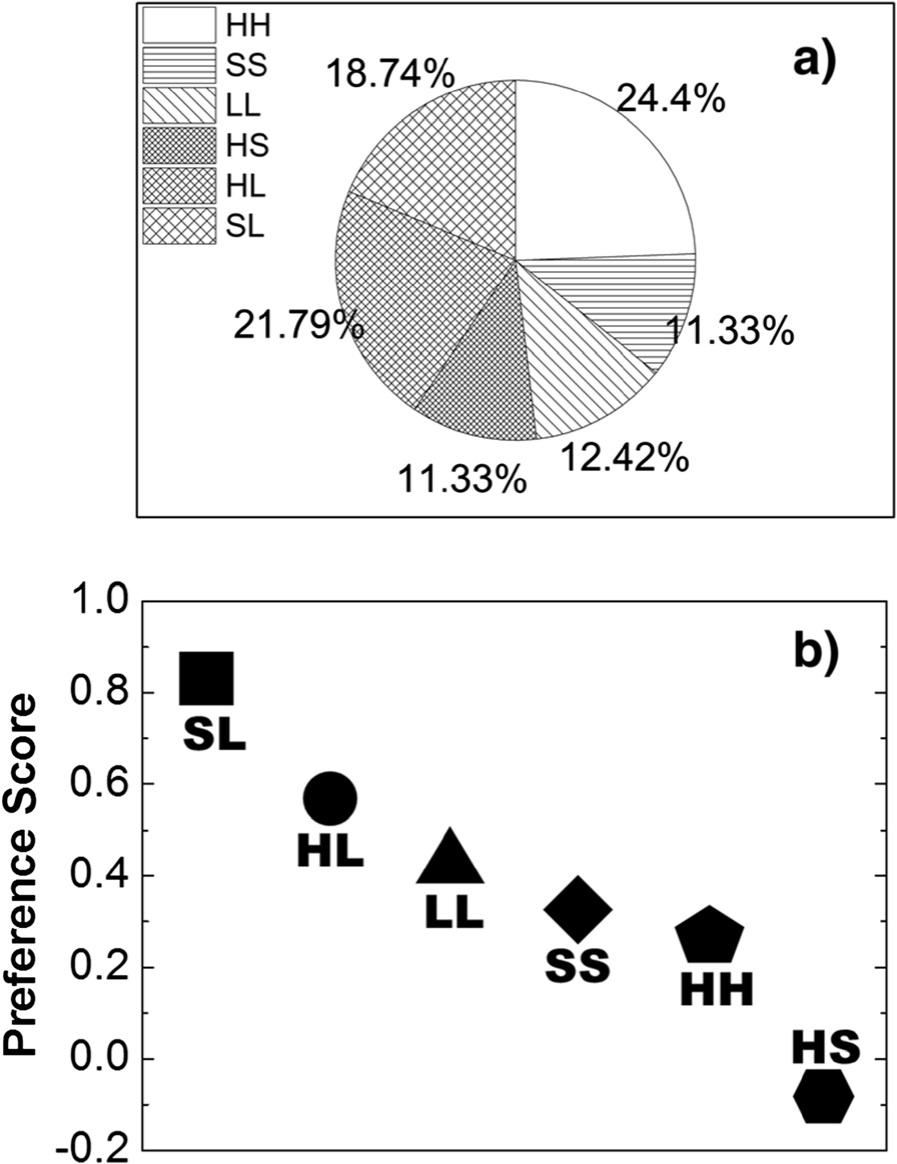
**Each fragment was divided into three categories, depending on the secondary structure type of the residue at the center of the fragment.** Consequently, the interface fragment pair was classified into six motifs. The percentage of these six motifs for all the 459 fragment pairs in the library is plotted in **(a)**. We further defined a preference score for each motif. A higher score for a specific motif indicates that it is more favored to form. Consequently, the preference scores for all six motifs are plotted in **(b)**. The figure suggests that fragment pair motifs are not equally distributed, but have strong preference.

We further investigated the distribution of protein secondary structures in the 459 representative models of interface fragment pairs. Each fragment in a pair was first divided into three categories: helix (H), strand (S) and loop (L). The criteria that each fragment belongs to one of these three categories depend on the secondary structure type of the residue at the center of the corresponding fragment. The secondary structure type of a residue is determined by the standard DSSP algorithm [34]. After we assigned categories for both fragments in a pair, the interface fragment pair can therefore be classified into the following six motifs: HH (a pair between two H fragments); SS (a pair between two S fragments); LL (a pair between two L fragments); HL (a pair between H and L fragments); HS (a pair between H and S fragments); SL (a pair between S and L fragments); The percentage of these six motifs is plotted in Figure 4a, after we got the secondary structure information for all the 459 fragment pairs in the library. The figure shows that these six motifs are not equally distributed in the library. For instance, the HH motif is more abundant than other motifs. In order to study the secondary structure preference of interface fragment pairs, the observed frequency of each motif need to be normalized by the probability of each secondary structural type at binding interfaces. Therefore, we calculated the distribution of H, S and L fragments in the chosen domain interfaces of the 3did database. The probability of H fragments appears at domain interfaces is 0.432. The probability of S fragments is 0.286, and the probability of L fragments is 0.282. We further defined a preference score for each motif. For instance, the preference score for HS motif is calculated as ln(*P*(*HS*)/(*P*(*H*)*P*(*S*))), in which P(HS) is the probability of finding HS motif at binding interfaces, and P(H) is the probability of finding H fragments at binding interfaces. A higher score for a specific motif indicates that it is more favored to form. Consequently, the preference scores for all six motifs are plotted in Figure 4B. Figure 4B shows that although HH motif is the most abundant motif in the library, its preference score is not the best, due to the highest probability of H fragment in the database. In contrast, loops are more preferred to appear at binding interfaces. Moreover, L fragments prefer forming heterogeneous contacts with S or H fragments. Finally, the interaction between H and S fragments is the least favored pattern.

**Figure 5:**
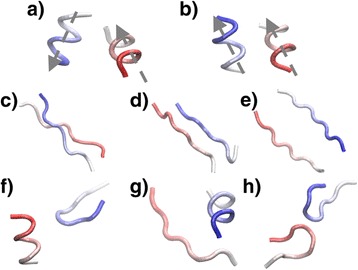
**We plotted the structures of some typical interface fragment pairs that are the most abundant in the library.** The two most abundant clusters are shown in **(a)** and **(b)**, both of which are HH motif. The direction of each α-helix is indicated by the arrow in the figures. Moreover, the interface fragment pairs from the SS motif are shown in **(c)**, **(d)** and **(e)**. Two fragments are either from a same piece of β-sheet that are connected by hydrogen bonds, or from two β-sheets facing each other. Finally, fragment pairs involving loops are shown in **(f)**, **(g)** and **(h)**.

Figure 5 shows the typical structures of some interface fragment pairs that are the most abundant in the library. The fragment from one protein domain is in red, while the fragment from the other domain in the complex is in blue. These structures are selected from different motifs. The two fragment pairs of HH motif are plotted in Figure 5a and b. These are the two most abundant structures in the library. The direction of each α-helix is indicated by the arrow in the figures. The packing of two interacting α-helices in these two fragment pairs form supplementary angle. If the binding interface from a pair of interacting proteins is located at their helical regions, Figure 5a and b suggest two favorite model of the complex’s quaternary structure. Moreover, the interface fragment pairs from the SS motif are shown from Figure 5c to e. Two modes are obtained if β-strands are co-localized at the binding interface. They are either from a same piece of β-sheet that are connected by hydrogen bonds, as shown in Figure 5c (parallel) and 5d (antiparallel), or from two β-sheets facing each other, as shown in Figure 5e. Finally, fragment pairs involving loops are shown from Figure 5f to h. They are structurally more diversified, but include less contact between two fragments than HH or SS motifs.

**Figure 6:**
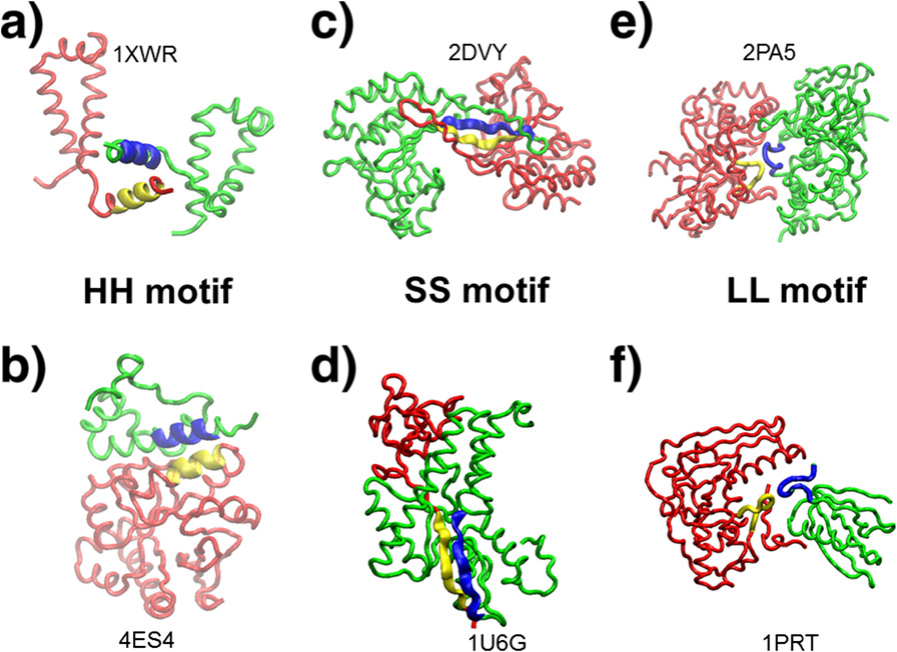
**Similar fragment pairs exist in different domain interactions.** Fragment pairs of two helices are located at the interfaces of two different domain interactions in **(a)** and **(b)**. Two fragments form inter-molecular β-sheet in **(c)** and **(d)**. One of these two structures is a homo-dimer and the other one is a heterodimer. Finally, similar LL motifs are found in very different binding interfaces, as shown in **(e)** and **(f)**. The backbones of interacting protein domains are in red and green, while the fragment pair motifs at their interfaces are in yellow and blue with cartoon representation.

Figure 6 illustrates that similar fragment pairs exist in different domain interactions. The backbones of interacting protein domains in the figure are in red and green, while the fragment pair motifs at their interfaces are in yellow and blue with cartoon representation. As shown in Figure 6a and b, fragment pairs of two helices are located at the interfaces of both dimers, while the structures of these two dimers are not identical. Furthermore, two fragments form inter-molecular β-sheet in both Figure 6c and d. One of these two structures, however, is a homo-dimer (Figure 6c) and the other one is a heterodimer (Figure 6d). Finally, similar LL motifs are also found in very different binding interfaces, as shown in Figure 6e and f. These popular fragment pairs indicate biological insights to protein-protein interactions. They reflect specific binding patterns which are significant to the cellular functions of proteins. For instance, death domain is the most important structural module involved in the regulation of apoptosis and inflammation. Packing between helices (Figure 5a and b) is the most common way in death domain induced complex assembly. Moreover, the SS motifs (Figure 5c and d) lead to the formation of intermolecular β-sheet. It is the major driven force of fibrous protein aggregations. Abnormal accumulation of these aggregates, known as amyloid fibrils, in organs may lead to amyloidosis, and may play a role in various neurodegenerative disorders. Finally, fragment pairs involving loops are the most common binding patterns of cell signal transduction, such as the binding motifs found in SH2 or SH3 domains.

### The benchmark test of complex assembly using interface fragment pair library

**Figure 7:**
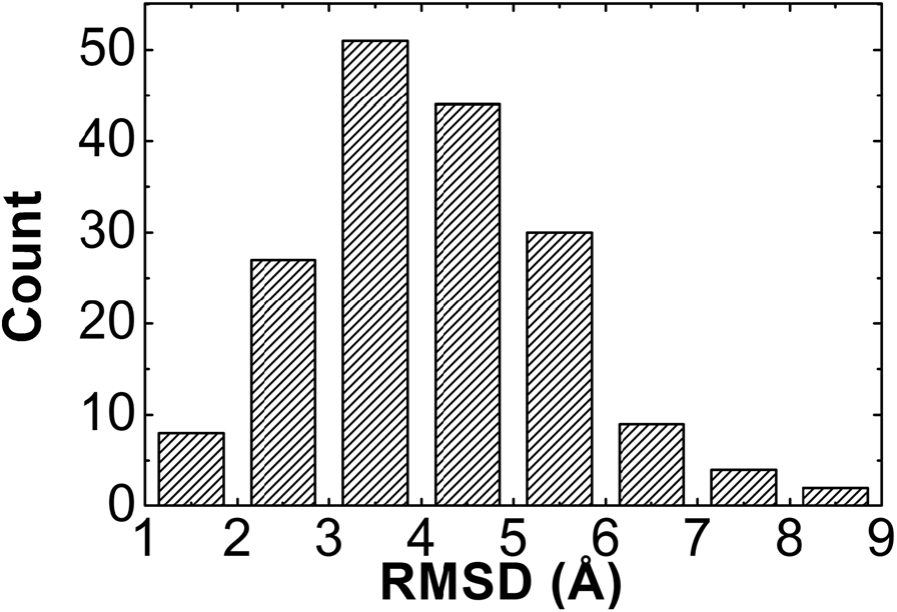
**We tested the library by a large-scale benchmark including a set of 176 non-redundant protein–protein complexes.** For each entry in the benchmark, a large number of structural models were generated. Among the derived ensemble of all complex models, we further found the target that has the lowest RMSD from the structure of the native complex. The distribution of the lowest RMSD models for all 176 entries is plotted by the histogram. The figure suggests that the native-like binding is highly likely in the structural ensemble of modeled protein complexes that were built through the library.

In order to evaluate the completeness of protein quaternary structural space represented by the interface fragment pair library, we applied the library to a large-scale benchmark set. The protein-protein docking benchmark constructed by ZLAB was used in our study [32]. The most updated version of the benchmark (4.0) includes a set of 176 non-redundant protein–protein complexes. For each entry in the benchmark, we first separated subunits from the complex. Subunits were assembled together by aligning the corresponding fragments in their structures with each of the 459 interface fragment pairs in the library, based on the algorithm introduced in the method. Among the derived ensemble of all complex models, we further found the target that has the lowest RMSD from the structure of the native complex. The distribution of the lowest RMSD models for all 176 benchmark entries is plotted as a histogram in Figure 7. The figure shows that the peak of the distribution is at 4.0 Angstrom. For more than 90% of the 176 entries, we can find structural models that have RMSD less than 6.0 Angstrom from the native complexes, indicating that the native binding can be reproduced with a high successful rate. Our benchmark results thus suggest that the space of protein quaternary structures can be simplified by a limited number of modes expanded by the interface fragment pair library. It is worth mentioning that the purpose of this test is not for systematic comparison of docking algorithms, but to enumerate all binding modes of a complex through a fragment-based library. Thereby, we used bound structures of subunits during complex assembly instead of unbound structures that are normally used in docking tests.

**Figure 8:**
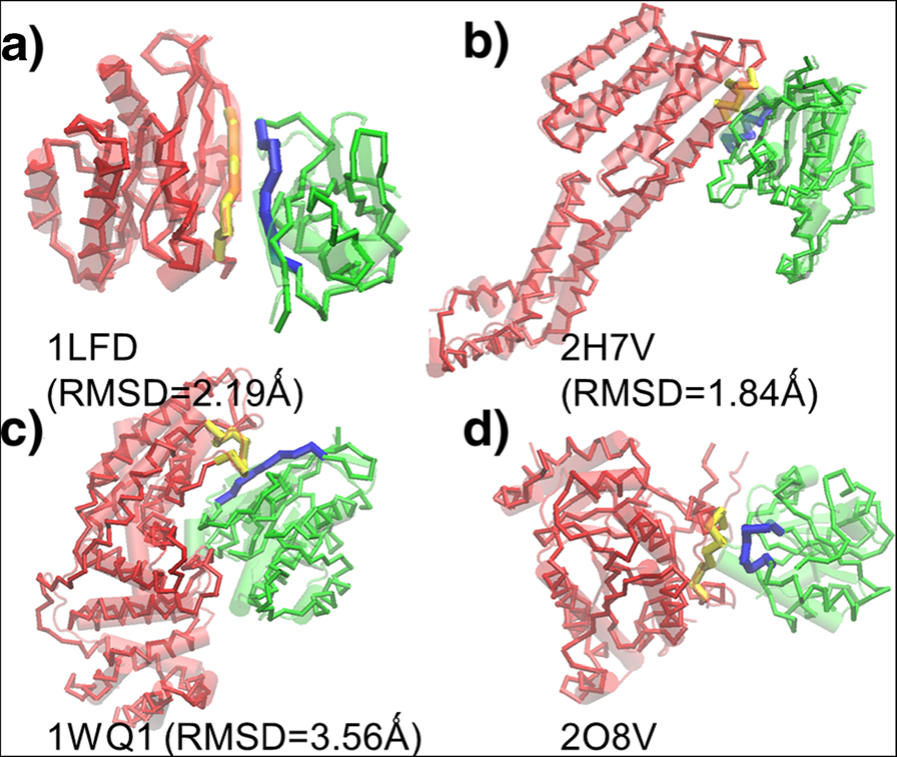
**We show some specific examples of our benchmark results.** A variety of different secondary structure types are presented at the binding interfaces of these complexes. An SS motif in which two strands form hydrogen-bond-based contacts is observed in **(a)**. A pair of helices is observed at the interface of **(b)**. Comparatively, the complex in **(c)** contains a more extensive interface. The native-like binding modes were successfully reproduced in these three cases, while **(d)** shows an example in which we failed to assemble the proteins into a complex that is close to the native quaternary structure. The interface fragment pairs are highlighted in the modeled structural complexes. The fragments located at the interfaces of green monomers are shown in blue and the fragments located at the interfaces of red monomers are shown in yellow.

Some specific examples of our modeling results are shown in Figure 8. The Cα traces in red and green are the lowest RMSD structural models of assembled receptors and ligands, while their native structures are superimposed transparently by cartoon representation. The PDB id of the selected complexes, and the RMSD values between the model and the native structures are also listed in the figure. A variety of different secondary structure types are presented at the binding interfaces of these complexes. An SS motif in which two strands form hydrogen-bond-based contacts is observed in Figure 8a, while in Figure 8b, binding is achieved through interactions between a pair of helices. Comparatively, the complex in Figure 8c contains a more extensive interface. In all these three cases, the native-like binding modes exist in the structural ensemble we constructed through the interface fragment pair library. In contrast, Figure 8d shows an example in which we failed to assemble the proteins into a complex that is close to the native quaternary structure. The interface of the receptor in this complex contains a long region of disordered loop. The corresponding fragment pairs in this interface may not appear in our library, leading into the result that its native-like binding cannot be derived.

In order to estimate the difficulty in finding good candidates from the ensemble of structural model, we have also included the total number of structural models generated for each entry in the benchmark in additional to the lowest RMSD. The distribution of total number of structural models for all 176 benchmark entries is plotted as a histogram in supplemental document as Additional file 1: Figure S2. The figure shows that our assembly algorithm generated less than 200 structural models for about 90% of entries. The average number of structural models over all entries is 127. The number of structural models generated in the ensemble of each entry depends on the size of interacting proteins, as well as the structural features at their binding interface. Overall, the result indicates that native-like binding modes between proteins can be found among a relatively small number of structural models by our assembling algorithm. Finally, it is worth mentioning that the library of 459 fragment pairs is a highly selective representation of the 3did database, which originally includes 153127 pairs. A clustering procedure was performed, and all pairs in one cluster were merged into one as a representative model. Additionally, only the most abundant clusters containing more than 20 members were selected. Consequently, the original information of which fragment pair belongs to which protein structure has been averaged our during this clustering and selection process. Furthermore, our current library of fragment pairs only contains structural information. There is no sequence information associated with these fragment pairs. In other words, the primary purpose of this study is to study the features of protein quaternary structural space. Therefore, in current benchmark test, we did not eliminate the potential overlap between the 3did database and the docking benchmark. However, in future development, statistical-based sequence profile will be assigned to fragment pairs in the library. The library with sequence information will be used to predict and evaluate the structural models of protein-protein interactions. Under this circumstance, the overlap between our library and any benchmark set will be accordingly removed.

## Conclusions

The physical interactions between proteins play pivotal roles in many biological processes. Understanding the structural features of these interactions is the basis to study protein functions in cells. However, the spatial arrangement between two interacting proteins or protein domains is highly diversified, leading into an interesting question of whether the complexity of protein binding interfaces can be simplified. Similar to the space of protein tertiary structures in which discrete patterns are clearly observed on fold and sub-fold motif levels, it has been found that the space of protein quaternary structures is highly degenerate due to the packing of compact secondary structure elements at interfaces. Therefore, it is necessary to further decompose the protein quaternary structural space into a more local representation. Fragment-based methods have been proved their success in predicting protein tertiary structures. In this article, similar idea has been extended to protein interfaces. Specifically, a library was constructed by collecting the interface fragment pairs from the structural database of protein interacting domains. After structural-based clustering, we found that more than 90% of these interface fragment pairs can be represented by a limited number of highly abundant motifs. These motifs were further used to guide complex assembly. A large-scale benchmark test shows that the native-like binding is highly likely in the structural ensemble of modeled protein complexes that were built through the library. Overall, our study presents supportive evidences that the space of protein quaternary structures can be represented by the combination of a small set of secondary-structure-based packing at binding interfaces.

In order to conduct a comparative study between different databases, we downloaded information of protein interactions from iPfam. The iPfam database includes a total number of 8160 intermolecular domain interactions. These interactions belong to either homodomain or heterodomain. For each interacting domain structure in iPfam, we enumerated all interface fragment pairs based on the same criteria introduced in the method. We compared all these interface fragment pairs with our fragment pair library derived from 3did. We calculated RMSD between fragment pairs in iPfam and our library. The RMSD of the closest fragment pair in the library was recorded. The distribution of this closest RMSD for fragment pairs in all 8160 iPfam interactions is plotted as a histogram in the supplemental document (Additional file 1: Figure S3). Based on the statistical results shown in the figure, for more than 98% of fragment pairs in iPfam database, we are able to find an entry from our library which RMSD is below 4 Angstrom. This comparative analysis indicates that the features of quaternary packing are conserved across different structural databases of protein-protein interactions. Moreover, the cutoff value of 20 members in each cluster was empirically determined. In order to test the robustness of our clustering procedure and investigate if change of this parameter does not significantly affect the quality of the final library, we reduced the cutoff value from 20 to 10, so that any clusters with larger than 10 members was selected. Consequently, the library was expanded from 459 to 596 clusters. We tested the library including 596 clusters to all domain interactions in the iPfam database. As shown in Additional file 1: Figure S3, the striped bar is the statistical results for library with 596 clusters, while black bar is the statistical results for the original library with 459 clusters. The figure shows that distributions in these two histograms are highly similar, indicating that changing cluster size does not significantly affect the quality of the final library.

Although the native-like complex models are among the structural ensembles in most cases of the benchmark test, as shown in Figure 7, we are not able to identify them from other models due to the fact that the current library is purely geometric-based. The primary purpose of constructing the library is not to develop a docking algorithm or predict protein-protein interactions, but to study the features of protein quaternary structural space. However, the method will become practically useful after integrating the sequence or energetic information into the library. For instances, future improvements include assigning statistical-based sequence profiles to each fragment pair in the library [35]. During complex assembly, corresponding sequences in proteins will be aligned to the profiles of the fragments. Only fragments with alignment scores higher than certain cutoff values will be selected. This will narrow down the searching space when we generate structural ensembles of target complexes. Moreover, our method can also be combined with binding sites prediction [36,37], so that only the fragments at the predicted binding sites of target proteins will be selected and compared with fragment pairs in the library. Finally, currently available energy-based or empirical scoring functions [38] can be applied to distinguish native-like conformations from generated structural ensembles. The interface fragment pair library provides an efficient tool of sampling the space of protein quaternary structures.

## Additional file

Additional file 1: Figure S1. We have tested the stability of our clustering process across different runs. Five independent clustering runs were carried out. Each run was generated by a random order. Clusters in all runs were ranked by the number of their members. The ranking profiles are plotted. The high similarity of these profiles suggests that the topology of clusters does not change between different runs. **Figure S2.** In order to estimate the difficulty in finding good candidates from the ensemble of structural model, we included the total number of structural models generated for each entry in the benchmark in additional to the lowest RMSD. The distribution of total number of structural models for all 176 benchmark entries is plotted as a histogram. The figure shows that our assembly algorithm generated less than 200 structural models for about 90% of entries. The average number of structural models over all entries is 127. The number of structural models generated in the ensemble of each entry depends on the size of interacting proteins, as well as the structural features at their binding interface. **Figure S3.** We calculated RMSD between fragment pairs in iPfam and our libraries. The RMSD of the closest fragment pair in each library was recorded. The distributions of this closest RMSD for fragment pairs in all 8160 iPfam interactions are plotted as histograms. The black bar is the statistical results for library with 459clusters, while the striped bar is the statistical results for library with 596 clusters. **Table S1.** We have tested the stability of our clustering process across different runs. Five independent clustering runs were carried out. Each run was generated by a random order. The clustering results are listed in the table.
