# Supplementary material for: Decomposing the space of protein quaternary structures with the interface fragment pair library
Source: BMC Bioinformatics. 2015 Jan 16;16(1):14. doi: 10.1186/s12859-014-0437-4 (PMC4384354; doi:10.1186/s12859-014-0437-4)
Supplement: Additional file 1: Figure S1. — We have tested the stability of our clustering process across different runs. Five independent clustering runs were carried out. Each run was generated by a random order. Clusters in all runs were ranked by the number of their members. The ranking profiles are plotted. The high similarity of these profiles suggests that the topology of clusters does not change between different runs. Figure S2. In order to estimate the difficulty in finding good candidates from the ensemble of structural model, we included the total number of structural models generated for each entry in the benchmark in additional to the lowest RMSD. The distribution of total number of structural models for all 176 benchmark entries is plotted as a histogram. The figure shows that our assembly algorithm generated less than 200 structural models for about 90% of entries. The average number of structural models over all entries is 127. The number of structural models generated in the ensemble of each entry depends on the size of interacting proteins, as well as the structural features at their binding interface. Figure S3. We calculated RMSD between fragment pairs in iPfam and our libraries. The RMSD of the closest fragment pair in each library was recorded. The distributions of this closest RMSD for fragment pairs in all 8160 iPfam interactions are plotted as histograms. The black bar is the statistical results for library with 459clusters, while the striped bar is the statistical results for library with 596 clusters. Table S1. We have tested the stability of our clustering process across different runs. Five independent clustering runs were carried out. Each run was generated by a random order. The clustering results are listed in the table. [file 12859_2014_437_MOESM1_ESM.doc]

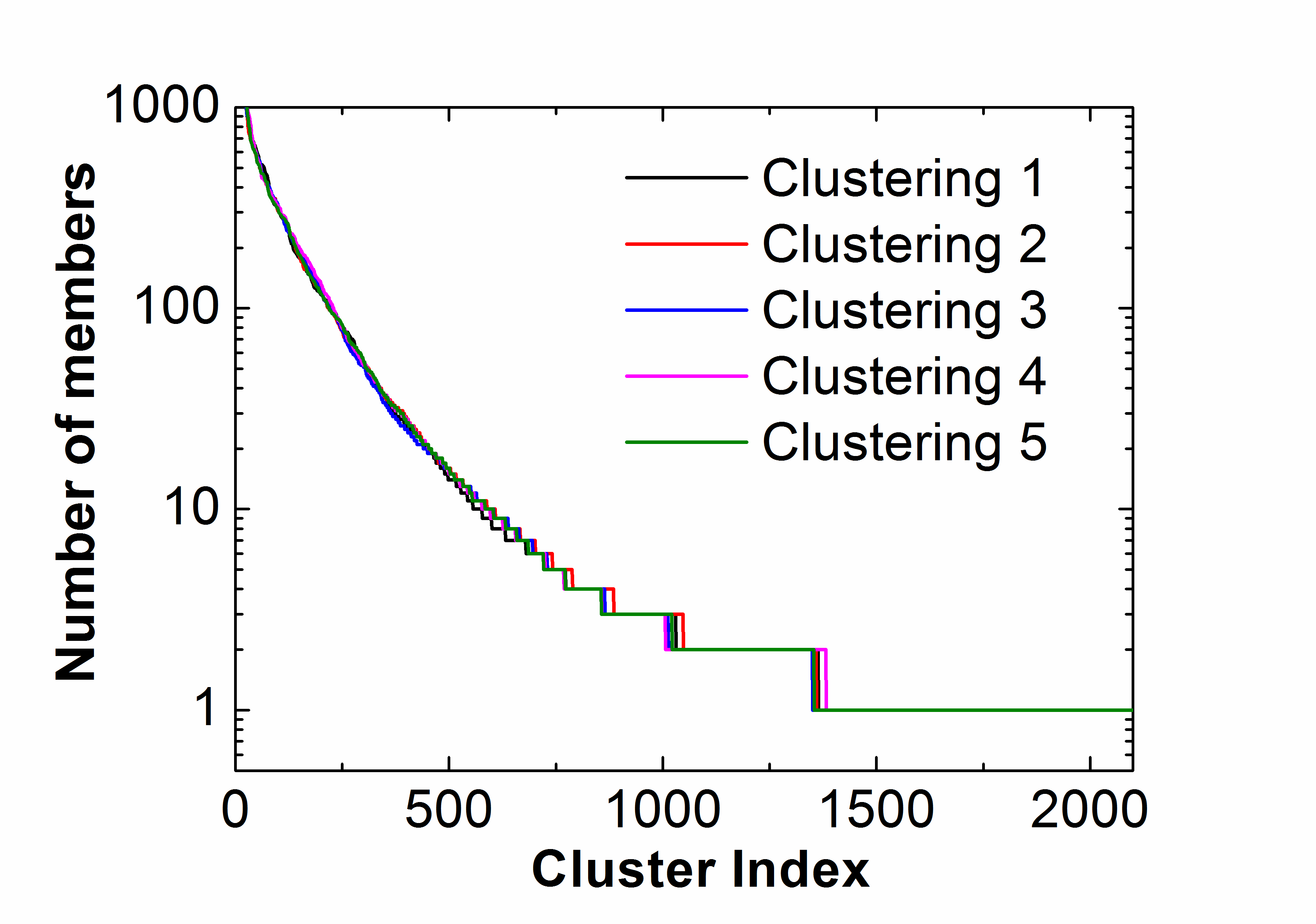


**Figure S1**

We have tested the stability of our clustering process across different runs. Five independent clustering runs were carried out. Each run was generated by a random order. Clusters in all runs were ranked by the number of their members. The ranking profiles are plotted. The high similarity of these profiles suggests that the topology of clusters does not change between different runs. These testing results indicate the stability of our clustering process.


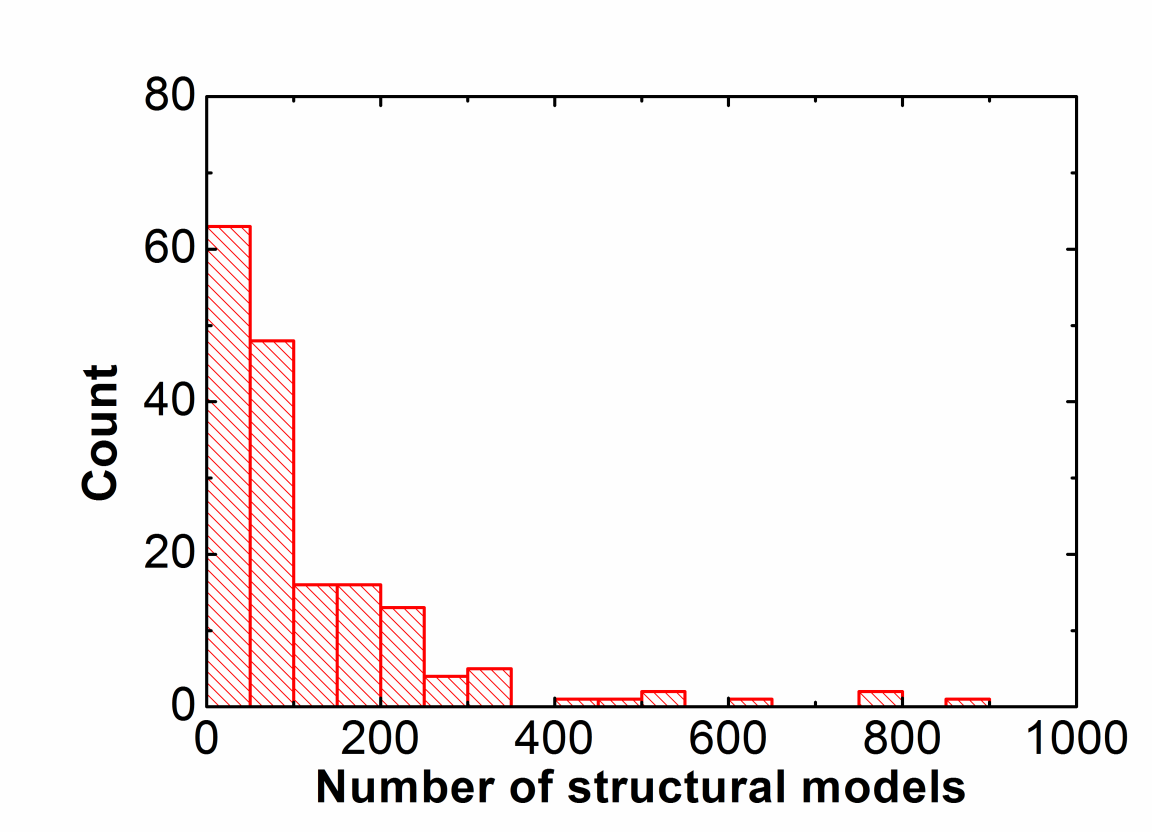


**Figure S2**

In order to estimate the difficulty in finding good candidates from the ensemble of structural model, we included the total number of structural models generated for each entry in the benchmark in additional to the lowest RMSD. The distribution of total number of structural models for all 176 benchmark entries is plotted as a histogram. The figure shows that our assembly algorithm generated less than 200 structural models for about 90% of entries. The average number of structural models over all entries is 127. The number of structural models generated in the ensemble of each entry depends on the size of interacting proteins, as well as the structural features at their binding interface. Overall, the result indicates that native-like binding modes between proteins can be found among a relatively small number of structural models by our assembling algorithm.


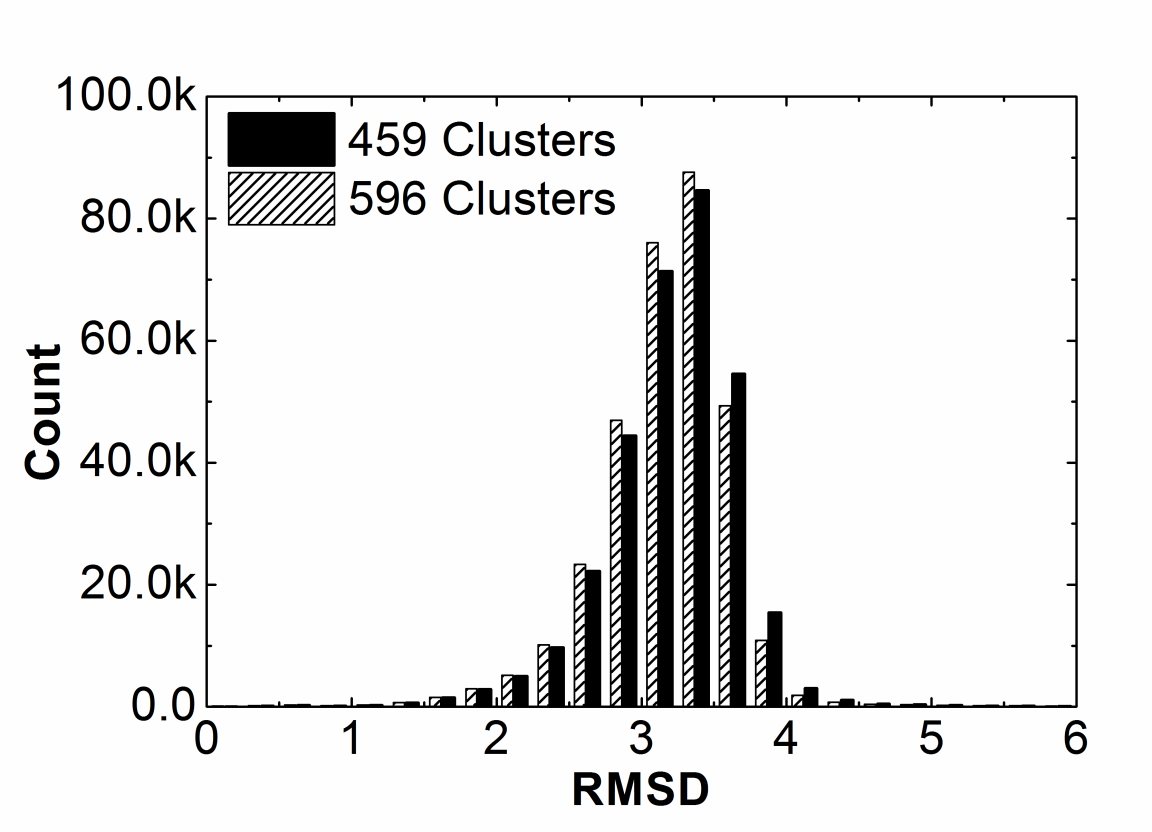


**Figure S3**

The cutoff value of 20 members in each cluster was empirically determined. In order to investigate if change of this parameter does not significantly affect the quality of the final library, we reduced the cutoff value from 20 to 10, so that any clusters with larger than 10 members was selected. Consequently, the library was expanded from 459 to 596 clusters. We tested both libraries including 459 and 596 clusters to all domain interactions in the iPfam database. Specifically, for each interacting domain structure in iPfam, we enumerated all interface fragment pairs based on the same criteria introduced in the method. We compared all these interface fragment pairs with both 459 and 596 fragment pair libraries. We calculated RMSD between fragment pairs in iPfam and our libraries. The RMSD of the closest fragment pair in each library was recorded. The distributions of this closest RMSD for fragment pairs in all 8160 iPfam interactions are plotted as histograms. The black bar is the statistical results for library with 459 clusters, while the striped bar is the statistical results for library with 596 clusters. The figure shows that distributions in these two histograms are highly similar, indicating that changing cluster size does not significantly affect the quality of the final library.

| Index | Total clusters | Clusters with more than 20 members |
| --- | --- | --- |
| 1 | 2135 | 459 |
| 2 | 2139 | 454 |
| 3 | 2131 | 449 |
| 4 | 2132 | 456 |
| 5 | 2125 | 459 |

Table S1

We have tested the stability of our clustering process across different runs. Five independent clustering runs were carried out. Each run was generated by a random order. The clustering results are listed in the table. All five runs ended up with very close number of total clusters. The numbers of the most abundant clusters which contain more than 20 members are also very close among these five runs.
